# A self-powered, flexible ultra-thin Si/ZnO nanowire photodetector as full-spectrum optical sensor and pyroelectric nanogenerator

**DOI:** 10.3762/bjnano.11.145

**Published:** 2020-10-27

**Authors:** Liang Chen, Jianqi Dong, Miao He, Xingfu Wang

**Affiliations:** 1 Institute of Semiconductor Science and Technology, South China Normal University, Guangzhou 510631, China; 2 School of Physics and Optoelectronic Engineering, Guangdong University of Technology, Guangzhou 510006, China

**Keywords:** flexible, full-spectrum, photodetector, pyroelectric nanogenerator (PENG), self-powered

## Abstract

In this work, a new type of self-powered, high-performance ultra-thin p-Si/n-ZnO nanowire (NW) flexible photodetector (PD) and its application as full-spectrum optical sensor and pyroelectric nanogenerator (PENG) are demonstrated. The working mechanism of PDs for PENGs is carefully investigated and systematically analyzed. The self-powered PDs exhibit high responsivity (1200 mA/W), high detectivity (10^13^ Jones) and fast response (τ_r_ = 18 μs, τ_f_ = 25 μs) under UV illumination. High and stable short-circuit output currents at each wavelength from ultraviolet (UV) to near-infrared (NIR) demonstrates that the device can realize full-spectrum optical communication. An experiment in which the PENG powers other devices is designed to further demonstrate the proposed working mechanism. This work provides an effective approach to realize self-powered, high-performance PDs for full-spectrum communication. Also, the fabrication of the PENG utilizing a simple and low-cost method shows its potential applications in self-powered flexible electronic devices.

## Introduction

Full-spectrum photodetectors (PDs) that can detect light from ultraviolet to near-infrared have attracted widespread attention in recent years for a variety of applications in industry and technology, such as optical sensing/communication, environmental monitoring, biomedicine, and the “internet of things” [1–4]. Especially full-spectrum PDs applied in flexible wearable electronic devices have been extensively researched [5]. The majority of the reported full-spectrum PDs, which are based on perovskites, quantum dots, or organic materials are costly, complicated to prepare, and difficult to integrate into flexible electronic devices. This limits their application in the field of flexible electronics. Recently, the design of flexible devices with Si membranes as building blocks has been explored. These devices have become promising candidates for the use as flexible PDs due to many advantages, such as good compatibility, working in a harsh environment, low cost, and easy preparation [6–9]. However, the energy supply system of traditional Si-based flexible PDs utilizes an external battery, which will affect the portability, comfort, and durability of wearable devices due to large volume, and limited capacity. Therefore, it is necessary to develop a new type of full-spectrum flexible PDs with a special working mechanism to further improve their performance and expand the practical application.

Pyroelectric polarization can be achieved by changing the temperature of a pyroelectric material [10]. ZnO is an ideal pyroelectric material with a wide bandgap (3.2 eV), which absorbs UV light and can be easily prepared [11–12]. A pyroelectric potential will be generated in ZnO when the temperature changes upon illumination. The internal electric field can effectively drive the flow of electrons through an external circuit, yielding a short-circuit current output that can be used as a power generator [13–14]. Pyroelectric nanogenerators (PENGs) based on a pyroelectric material have been demonstrated as an effective approach that could avoid the waste of energy by converting temperature fluctuations induced by photoabsorption into electric energy. This is achieved by utilizing the temperature dependence of electric displacement of polar materials, which are already applied in solar cells, photodetectors, temperature sensors, and stretchable electronics [15–18]. It means that the PENGs could serve as a power source to power PDs by harvesting energy from the working environment instead of a battery. Comparing with another emerging nanogenerators (triboelectric nanogenerators) [19–20], the PENGs benefit from not requiring external mechanical energy and can make full use of the energy in their own environment. This self-powered system can greatly improve the portability and durability of the flexible PDs.

In this work, a new type of self-powered high-performance full-spectrum flexible PDs consisting of ultra-thin p-Si/n-ZnO nanowires (NWs) is fabricated. The working mechanism of PDs based on p-Si/n-ZnO heterojunctions for PENGs is carefully investigated and systematically analyzed. Also, the impact of the periodic frequency of the illumination and the optical power density on the short-circuit current and performance of PDs is analyzed carefully. This self-powered PDs show a full-spectrum response range from UV (325 nm) to near-infrared (NIR) (1064 nm) under zero bias with fast response speed at each wavelength. The self-power PDs exhibit high responsivity (1200 mA/W), high detectivity (10^13^ Jones) and fast response speed (τ_r_ = 18 μs, τ_f_ = 25 μs) under UV illumination. The pyroelectric output current can drive a LED by harvesting thermal energy induced by photoabsorption. This work provides an effective approach to realize self-powered, high-performance full-spectrum PDs utilizing a simple and low-cost method.

## Results and Discussion

### Preparation and characterization of the flexible PDs

**Figure 1:**
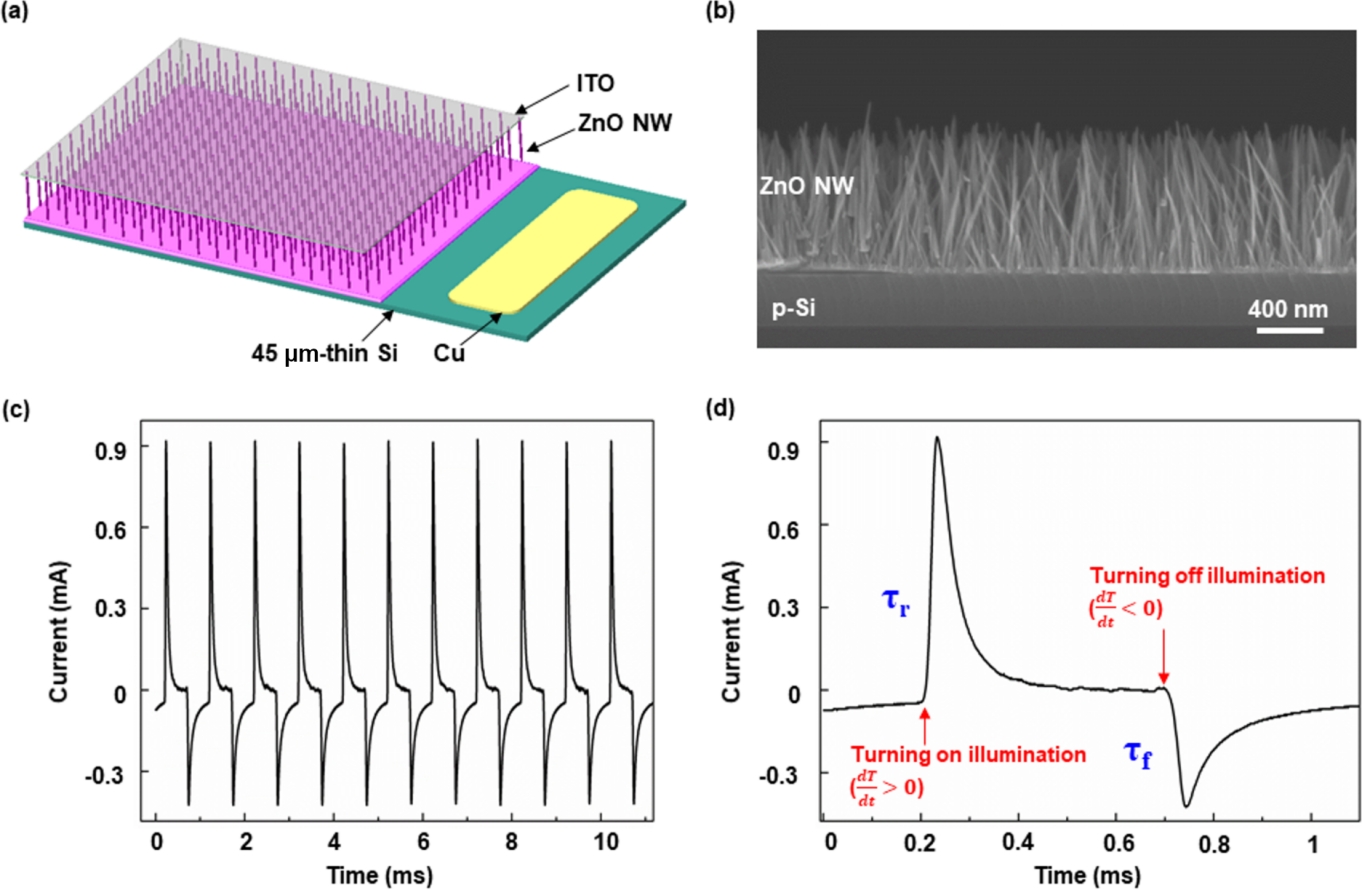
(a) Structure diagram of p-Si/n-ZnO NWs PDs. (b) Cross-sectional SEM image of the as-grown Si/ZnO NWs heterojunction. (c) Dynamic response curve *I–t* under 325 nm ultraviolet illumination and zero bias. (d) One typical cycle of short-circuit *I–t* curve.

The schematic of a self-powered full-spectrum PD based on a p-Si/n-ZnO NWs heterojunction is illustrated in Figure 1a. A typical cross-sectional scanning electron microscopy (SEM) image of the as-grown Si/ZnO NWs heterojunction is shown in Figure 1b. The uniformly grown ZnO NWs are conducive to a stable short-circuit current output. The detailed process of device preparation is shown in the Experimental section. The 45 μm ultrathin p-Si layer is obtained by isotropic chemical etching to realize a flexible device. Importantly, a previous study has shown that the performance of PDs (regarding, e.g., transient current and response speed) of PDs is significantly improved by reducing the thickness of Si [21]. In order to investigate the performance of self-powered PDs, a dynamic response curve *I*–*t* was measured under periodic 325 nm ultraviolet (UV) illumination and zero bias (Figure 1c). The photoresponsivity is calculated by Equation 1 [21]:

[1] $$R=\frac{i_{\text{py}}-i_{\text{dark}}}{P_{\text{in}}},$$

where *i*_py_, *i*_dark_, and *P*_in_ represent the pyroelectric current, the dark current, and the input power, respectively. The calculated photoresponsivity *R* is up to 1200 mA/W, which is more ten times than that of Perovskite/ZnO and Si/ZnO PDs [22–23]. The detectivity D* is one of the key parameters of a PD, which usually describes the ability to detect weak signals. The photoresponsivity is calculated by Equation 2 [3]:

[2] $$D^{*}=R{\left(\frac{S}{2qI_{\text{dark}}}\right)}^{1/2},$$

where *R*, *S*, *q*, and *I*_dark_ represent responsivity, illuminated area, electronic charge, and dark current, respectively. The calculated detectivity D* of our device is as high as 10^13^ Jones. A one-cycle dynamic response curve extracted from Figure 1c is shown in Figure 1d, indicating that the PD possesses a fast response speed (rising time τ_r_ = 18 μs, falling time τ_f_ = 25 μs). It is obvious that the p-Si/n-ZnO NWs heterojunction PDs exhibit excellent detection capability and work well without an external power source.

### Working mechanism of a PENG based on p-Si/n-ZnO NWs heterojunctions

**Figure 2:**
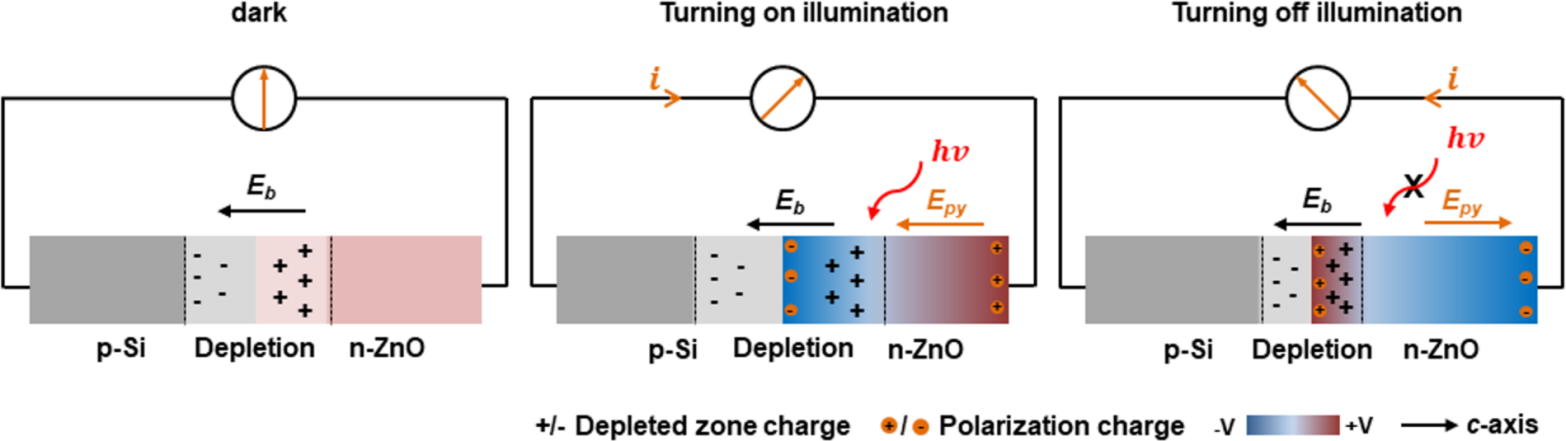
Working mechanism of PENGs. under zero bias, a depletion zone and corresponding built-in electric field are formed without illumination (left). Upon illumination, the pyroelectric electric field and transient output current are generated (middle). Reverse pyroelectric field and reverse transient output current are generated at the moment of turning off illumination (right).

To illustrate the working mechanism of self-powered PDs, schematic diagrams for the different conditions are presented in Figure 2. In the dark, a depletion layer is formed at the heterojunction interface due to carrier diffusion, and a corresponding intrinsic electric field (*E*_b_) is formed in the depletion zone (Figure 2, left). Under this circumstance, the electron diffusion current and drift current are equal in magnitude and opposite in direction in the heterojunction. Therefore, the net current flowing through the heterojunction is zero, and no current flows through the external circuit. However, upon illumination, a photothermally induced instantaneous temperature increase (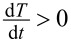) will lead to the generation of a pyroelectric polarization potential and pyroelectric polarization charges at both ends of the *c*-axis of the ZnO NWs (Figure 2, middle). Because the direction of the pyroelectric electric field (*E*_py_) is the same as *E*_b_ and the barrier height decreases at the heterojunction interface due to the generation of a negative polarization potential, the total electric field in the depletion zone increases instantly. The electric field in the depletion zone drives electrons to flow from Si to ZnO and produces a transient short-circuit output current in the external circuit. According to the pyroelectric theory, the pyroelectric current can be determined by Equation 3 [24]:

[3] $$i_{\text{py}}=PA\frac{\text{d}T}{\text{d}t},$$

where *P*, *A*, and *T* represent pyroelectric coefficient, effective area, and temperature, respectively. The corresponding open-circuit voltage can be determined by Equation 4 [25]:

[4] $$V_{\text{py}}=PA\frac{\Delta T}{C},$$

where *P*, *A*, *C,* and *T* represent pyroelectric coefficient, effective area, equivalent capacitance of device, and temperature, respectively. A large transient pyroelectric current occurs in the moment when the illumination is turned on (Figure 1d). When the illumination is turned off, the photothermallly induced instantaneous temperature decrease (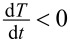) will lead to the generation of a pyroelectric polarization potential and pyroelectric polarization charges opposite to those before, when the illumination was turned on (Figure 2, right). The depletion zone width and corresponding electric field will decrease due to the generation of the reverse pyroelectric field (*E*_py_) in the opposite direction of *E*_b_. The total electric field in the depletion zone decreases instantly. The electric field drives electrons to flow from ZnO to Si and produces a transient short-circuit output current in the external circuit. Therefore, the device based on p-Si/n-ZnO NWs heterojunction can serve as a PENG, and the self-powered full-spectrum PD can work at zero bias without any power sources.

### Dependence of the short-circuit current on periodic frequency and power density of the illumination

**Figure 3:**
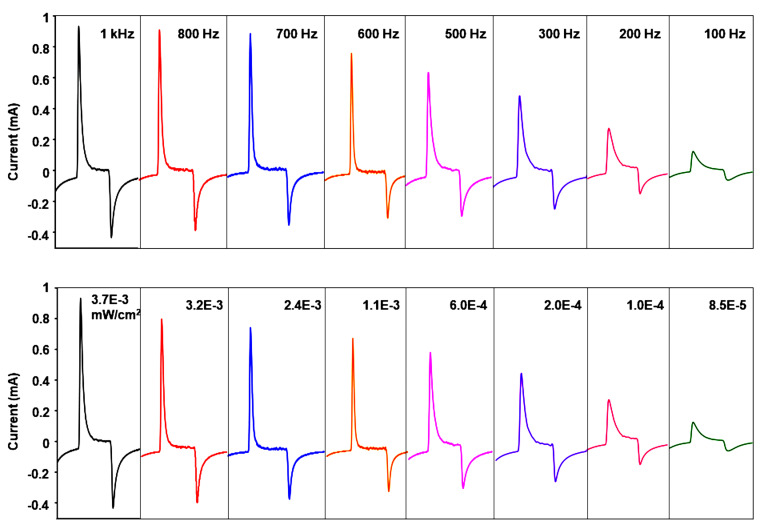
Impact of the incident optical power density and periodic frequency on the short-circuit current. *I–t* characteristics of a PD under zero bias and 325 nm UV illumination with different periodic frequencies ranges from 100 Hz to 1 kHz (top row). *I–t* characteristics of a PD under zero bias and 325 nm UV illumination with different power densities ranges from 8.5 × 10^−5^ to 3.7 × 10^−3^ mW/cm^2^ (bottom row).

The impact of UV illumination with different periodic frequencies and power densities on the performance of the self-powered PDs is studied and summarized carefully in Figure 3. The short-circuit current response of PDs to 325 nm UV illumination under different frequencies ranging from 100 Hz to 1 kHz is shown in the top row of Figure 3. Obviously, the short-circuit current increases with increasing the frequency, indicating that the higher the frequency, the stronger the pyroelectric effect and the pyroelectric current. The reason for this phenomenon is mainly that the pyroelectric current induced by the pyroelectric effect is proportional to 
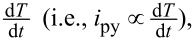
 that is, inversely proportional to Δ*t*. The short-circuit current response to UV illumination under zero bias is also measured and systematically investigated by varying the power density from 8.5 × 10^−5^ to 3.7 × 10^−3^ mW/cm^2^. The light-induced temperature difference Δ*T* increases with the increase of incident optical power density. Therefore, a larger short-circuit current will be generated because the pyroelectric current is proportional to Δ*T*. At the moment of turning off the illumination, the transient temperature decreases and the corresponding temperature change rate is less than zero (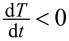). Therefore, there is an negative transient current. As in the case of turning on the illumination, the transient current gradually increases with the increase of periodic frequency. According to the above analysis, incident optical power density and periodic frequency play an important role in the performance of PDs and output current as a PENG.

### Full spectrum response

**Figure 4:**
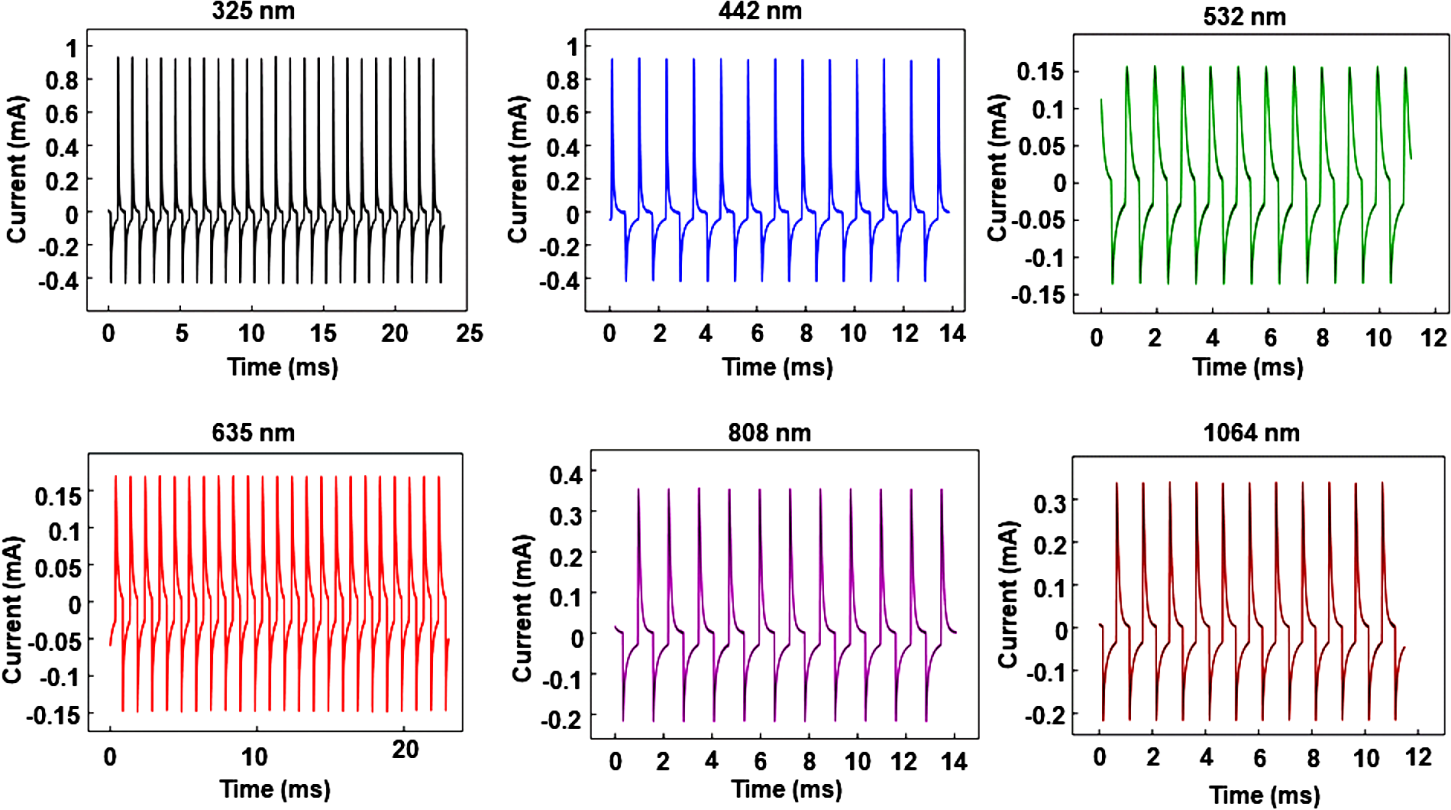
Dynamic response characteristics *I–t* characteristics of PDs under zero bias and different incident optical wavelengths in the range from UV to NIR.

To further investigate the performance of PDs in the illumination range from UV to NIR, the laser was operated at 325, 442, 532, 635, 808, and 1064 nm as an excitation source. The dynamic characteristic curves *I–t* of a PD under zero bias and at different laser wavelengths are shown in Figure 4. It is obvious that there are large short-circuit currents at all wavelengths, indicating that the self-powered PDs yields full-spectrum (UV–visible–NIR) detection. The broad spectral photoresponse from UV to NIR is related to two different bandgap materials (wide bandgap 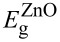 = 3.2 eV and narrow bandgap 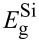 = 1.1 eV). Remarkably, the photoresponse current is significantly enhanced by introducing the pyroelectric effect based on the ZnO pyroelectric material. Moreover, response time and high sensitivity are almost the same at each wavelength, and the device exhibits excellent stability and repeatability in the UV–visible–NIR range. Therefore, the self-powered p-Si/n-ZnO NWs heterojunction device can be applied in full-spectrum optical sensing or optical communication.

### Powering external circuits as a PENG

**Figure 5:**
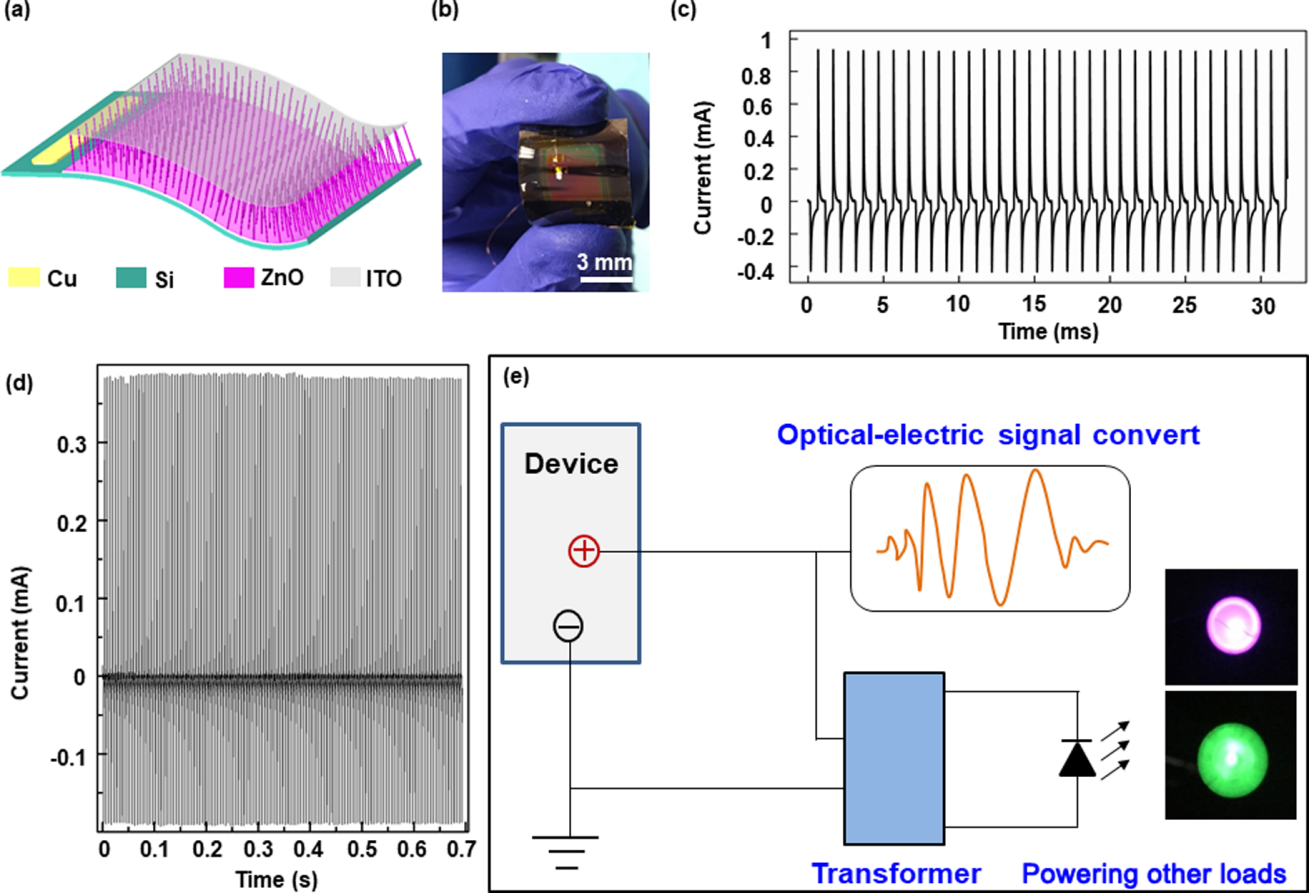
(a) Structure diagram of an ultra-thin (45 μm) p-Si/n-ZnO heterojunction device. (b) Optical image of the flexible PD. (c) Short-circuit photoresponse current of the ultra-thin (45 μm) p-Si/n-ZnO heterojunction device under zero bias by periodically turning on/off 325 nm UV illumination with a frequency of 1 kHz. (d) Dynamic response characteristics under 1064 nm NIR illumination. (e) Schematic diagram of a PENG powering different loads.

In structure design of the device, ultra-thin (45 μm) p-Si is prepared by isotropic chemical etching to fabricate flexible electronic devices and enhance the performance of the PDs. The structure diagram of a flexible device based on p-Si/n-ZnO NWs heterojunction is shown in Figure 5a, with a corresponding optical image of the sample shown in Figure 5b. The device can work well under repeated bending, ensuring the practicality of the device. The short-circuit output current to 325 nm UV illumination is measured under zero bias by periodically turning on/off illumination with a 1 kHz frequency as plotted in Figure 5c. A stable output is necessary for a power source to ensure the load can work stably. Moreover, the maximum output current is up to 1.5 mA with a corresponding voltage of up to 0.5 mV. The data about shown in Figure 5d indicates the stability and repeatability of device. To demonstrate the p-Si/n-ZnO NWs heterojunction PDs as a PENG can be used as a direct power source, a schematic diagram of the PENG powering the load is shown in Figure 5e. The output current through the transformer can be used to power LEDs (inset in Figure 5e). The rated power of the green LED is 0.06 W and the corresponding rated current is 20 mA. The rated power of the red LED is about 0.04 W and the corresponding rated current is also 20 mA. Based on the investigation of directly powering the LEDs, we can conclude that the p-Si/n-ZnO NWs heterojunction device not only can realize self-powered full-spectrum (UV–visible–NIR) optical sensing but can also serve as a power source (PENG) transforming thermal energy into electrical energy to power other loads.

## Conclusion

We have fabricated a high-performance self-powered flexible p-Si/n-ZnO NWs heterojunction PDs for full-spectrum optical sensing and as a pyroelectric nanogenerator. The working mechanism of PDs for PENGs is carefully investigated and systematically analyzed. By changing the periodic frequency and the power density of the illumination the short-circuit current and the performance of PDs are notably improved. The self-powered PDs exhibit high responsivity (1200 mA/W), high detectivity (10^13^ Jones) and fast response speed (τ_r_ = 18 µs, τ_f_ = 25 µs) under UV illumination. High and stable short-circuit output currents at each wavelength illumination range from UV to NIR demonstrate that the device can realize full-spectrum optical communication. The experiment in which the PENG powers other loads is designed to further demonstrate the proposed working mechanism. Therefore, this work provides an effective approach to realize self-powered, high-performance PDs for full-spectrum communication and PENGs utilizing a simple and low-cost method, which offer potential applications in self-powered flexible electronic devices.

## Experimental

**Fabrication process of the device:** Firstly, the ultra-thin (45 μm) p-Si substrate was prepared by isotropic chemical etching. More specifically, a 500 μm p-type high conductivity Si substrate was dipped into potassium hydrate (KOH) solution with a concentration of 50% at 130 °C for 6–8 h. Then, the obtained 45 μm p-Si was washed with acetone, isopropanol, and deionized water. Secondly, a thin ZnO seed layer was deposited onto 45 μm p-Si by radio frequency (RF) magnetron sputtering. Next, the sample was placed into a solution of 0.877 g hexamethylenetetramine, 1.372 g Zn(CH_3_COO)_2_, and 13 mL ammonium hydroxide to grow ZnO NWs for half an hour via a hydrothermal method in a mechanical convection oven at 90 °C. Finally, by RF magnetron sputtering, a 200 nm thick layers of ITO and Cu were deposited on ZnO NWs and p-Si, respectively.

**Electrical measurements:** the measurement setup includes a source meter, an optical platform, a chopper, sample, and a light source. Sample, chopper and light source must be in the same straight line. The voltage of source meter is set as 0 V. When controlling the frequency of the chopper, the incident light power density and the wavelength of the incident light, the transient response current, the different light power density, and the wavelength of the incident light are measured.

**Instruments:** The scanning electron microscope is a Hitachi SU8010. The *I–t* characteristic curves are measured by a source table (SR570, DS345). The optical input stimuli are provided by a He–Cd dual-color laser (MCLS1, Thorlabs Inc.). The light power density used in this work was measured and obtained by a thermopile power meter (Newport 818P-001-12).
